# Viral blips during suppressive antiretroviral treatment are associated with high baseline HIV-1 RNA levels

**DOI:** 10.1186/s12879-016-1628-6

**Published:** 2016-06-21

**Authors:** Erik Sörstedt, Staffan Nilsson, Anders Blaxhult, Magnus Gisslén, Leo Flamholc, Anders Sönnerborg, Aylin Yilmaz

**Affiliations:** Department of Infectious Diseases, Institute of Biomedicine, Sahlgrenska Academy, University of Gothenburg, 413 90 Gothenburg, Sweden; Department of Mathematical Sciences, Chalmers University of Technology, 412 58 Gothenburg, Sweden; Department of Infectious Diseases, Venhälsan-Södersjukhuset, 118 83 Stockholm, Sweden; Department of Infectious Diseases, Malmö University Hospital, 205 02 Malmö, Sweden; Department of Infectious Diseases, Karolinska Institute, Karolinska University Hospital, 141 86 Stockholm, Sweden; Department of Clinical Microbiology, Karolinska Institute, Karolinska University Hospital, 141 86 Stockholm, Sweden

**Keywords:** Viral blip, Transient viremia, HIV-1, Antiretroviral therapy

## Abstract

**Background:**

Many HIV-1-infected patients on suppressive antiretroviral therapy (ART) have transiently elevated HIV RNA levels. The clinical significance of these viral blips is uncertain. We have determined the incidence of blips and investigated important associations in the Swedish HIV-cohort.

**Methods:**

HIV-1-infected ART naïve adults who commenced ART 2007–2013 were retrospectively included. Viral blips were defined as a transient viral load between 50 and 500 copies/mL Subjects not suppressed after six months on ART were excluded.

**Results:**

Viral blips were found in 76/735 included subjects (10.3 %) and in 90/4449 samples (2.0 %). Median blip viral load was 76 copies/mL (range 56–138). Median follow-up time was 170 weeks (range 97–240). Baseline viral load was higher in subjects with viral blips (median log_10_ 4.85 copies/mL) compared with subjects without blips (median log_10_ 4.55 copies/mL) (*p* < 0.01). There was a significant association between viral blips and risk for subsequent virological failure (*p* < 0.001).

**Conclusions:**

The Swedish national HIV-cohort has a low incidence of viral blips (10 %). Blips were associated with high baseline viral load and an increased risk of subsequent virological failure.

## Background

Most patients on combination antiretroviral therapy (cART) reach the goal of therapy, HIV RNA < 50 copies/mL blood, within three to six months after initiation of cART [1, 2]. When quantified with more sensitive methods, HIV can usually be detected in low concentrations in almost all adherent patients on effective therapy. The level of this so-called residual viremia has been demonstrated to be between 1 and 10 copies/mL [3–5].

Even though the plasma viral load is suppressed to < 50 copies/mL in a majority of treated patients, it may increase to detectable levels from time to time, usually to a maximum of 500 copies/mL, before decreasing to < 50 copies/mL again, so called viral blips [6]. There may be several reasons for these viral blips, including technical errors or an influence of the type of blood collection tube used [7]. Other explanations could be stochastic variations [8], conditions that temporarily could lead to increased HIV replication such as infections [9] or vaccinations [10–12], and low drug concentrations in blood because of poor adherence or poor absorption of antiretroviral drugs. Prior studies have reported blip incidence between 13 and 40 % [13–16]. The large differences are due to different blip definitions and viral load assays.

Possible consequences of viral blips are not clear. Some studies have found a correlation between viral blips and subsequent virological failure [17–20], whereas others have not [8, 13, 14, 21–26]. The aim of this study was to analyse the occurrence of viral blips in Swedish HIV-1 infected individuals with modern cART, and to find out whether any predictive factors could be identified between viral blips and variables such as nadir CD4^+^ T-cell count, pre-treatment viral load, choice of cART, and some co-morbidities.

## Methods

As part of clinical care, > 99 % of HIV-1-infected individuals living in Sweden are followed from diagnosis and onwards using a clinical decision support tool, called InfCare HIV. Clinical data from InfCare HIV is transferred in real time to a research database. All demographic data, all HIV RNA levels, all CD4+ T-cell counts, all ART regimens the patient has ever been on, are registered in the database. In this retrospective study, we included subjects matching our inclusion criteria. Participants had to be HIV-1-infected adults (≥18 years old) receiving their first line of cART and they had to have been on treatment for at least six months with at least one HIV RNA value < 50 copies/mL to secure treatment response before data was collected. The included patients were from five of Sweden’s largest HIV-clinics (the Department of Infectious Diseases at Karolinska University Hospital, Stockholm, Venhälsan at Södersjukhuset, Stockholm, the Departments of Infectious Diseases and Dermatology at Sahlgrenska University Hospital, and the Department of Infectious Diseases at Malmö University Hospital). These five clinics account for approximately two thirds of all HIV-1-infected patients in Sweden. Subjects were followed from August 2007 to December 2013 in two clinics (596 patients), and from June 2009 to December 2013 in the remaining three clinics (139 patients). The different starting dates for inclusion was because the ethylene diamine tetraacetic acid (EDTA) test tubes and the highly sensitive COBAS TaqMan HIV-1 technique (CAP/CTM2; Roche, Molecular Systems, Branchburg, NJ, USA), with a lower detection limit of 40 and 20 copies/mL (version 1 and 2), respectively, were introduced at different time-points at the different clinics. All patients at these clinics are followed according to the Swedish guidelines for treatment of HIV-1 infection. After initiation of cART, plasma HIV RNA is followed after 3 and 6 months, and after that every third to sixth months depending on the patient and the clinician.

Viral blips were defined as transient plasma HIV RNA levels between 50 and 500 copies/mL preceded and followed by HIV RNA < 50 copies/mL. Two values within six weeks apart were interpreted as one isolated blip where the highest HIV RNA level was noted. Patients were excluded if they had a viral failure, defined as a single HIV RNA measurement > 500 copies/mL or two consecutive samples ≥ 50 copies/mL drawn more than six weeks apart. Treatment interruptions of shorter than one month caused a pause in data collection for six months from the reinitiation of ART. If the interruption was longer than one month no more data was analysed after the interruption. If the last registered sample was 50–499 copies/mL this data was excluded to avoid confusion between possible blips and viral failure.

Samples with HIV RNA levels between 20 and 50 copies/mL were registered separately unless they were taken within six weeks from a blip and thus interpreted as part of the transient viremia. Demographic and medical data were collected, and included country of origin, age, gender, source of transmission, ethnic background, date of diagnosis and start of treatment, treating clinic, baseline viral load (VL) sampled within one month before treatment initiation, CD4 nadir, baseline primary drug-resistance mutations, choice of cART, co-infections with hepatitis B and/or C, and the presence or not of AIDS-defining conditions. Treatment regimen was registered for each blood sample during the study period.

### Statistical analysis

Mann-Whitney and chi-square tests were used for group comparisons of continuous and categorical data, respectively. Descriptive continuous data are presented as median and interquartile range (IQR) while categorical variables are listed as numbers and percentages unless otherwise stated. Statistical analyses were performed using SPSS (IBM Corp. Released 2011. IBM SPSS Statistics for Windows, Version 20.0. Armonk, NY: IBM Corp.).

To deal with the repeated measurements we primarily used Generalized Estimation Equations (GEE) with a binary logistic link for both univariable and multivariable analysis. The association between blips and HIV RNA measurement in the range between 20 and 50 copies/mL, sometimes referred to as low-level viremia was evaluated by stratifying the subjects for the number of samples below 50 copies/ml and then randomly shuffling the blip indicator within each strata to achieve an empirical distribution from which the significance could be obtained. A similar procedure was used to evaluate the association between failure and blips.

## Results

**Fig. 1:**
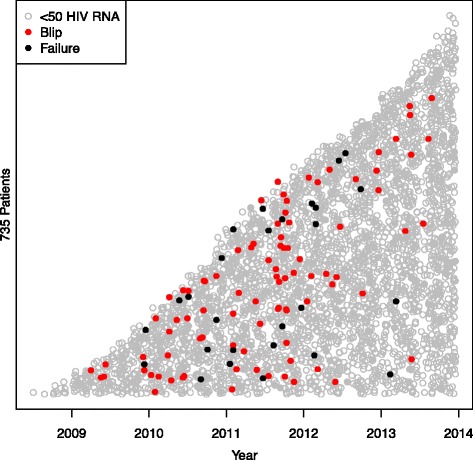
Figure 1 shows all 735 included patients and 4449 samples analysed. The y-axis represents all included patients on stacked horizontal lines (not included) depending on when they entered the study and the x-axis represents the study period. Each dot represents a blood sample. Grey empty circles show HIV RNA < 50 copies/mL, red filled circles indicate a blip between 50 and 500 copies/mL and black filled circles indicate viral failure

**Fig. 2:**
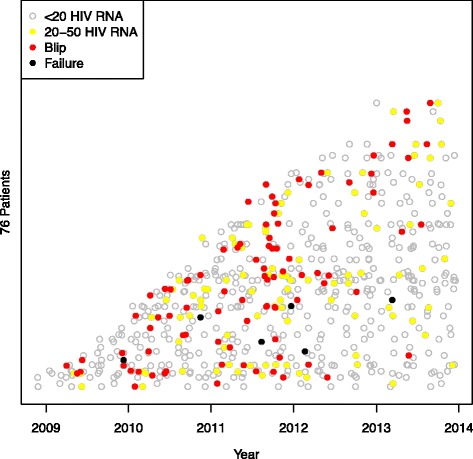
Figure 2 shows patients with blips. The y-axis represents the 76 patients with registered viral blips on stacked horizontal lines (not included) depending on when they entered the study and the x-axis represents the study period. Each circle represents a blood sample. Grey empty circles indicate HIV RNA < 20 copies/mL, yellow filled circles low-level viremia (20–50 copies/mL), red filled circles a blip between 50 and 500 copies/mL, and black filled circles signal viral failure

In total, 4449 blood samples from 735 HIV-1-infected individuals were included (515 men and 224 women, ages ranging from 22 to 81 years) (Fig. 1). Median follow-up time was 170 weeks (range 97–240). Both mean and median number of registered blood samples per patient was 6 (range 1–22). Seventy-six out of the 735 patients had at least one episode with transient viremia (Fig. 2), resulting in an incidence of 10.3 %. The majority of subjects (63/76) had one blip, twelve subjects had two blips, and one had three blips. HIV RNA levels during the blips ranged from 50 to 443 copies/mL (median 76). Patient characteristics are shown in Table 1.

**Table 1:** Patient baseline characteristics

	Transient viremia (blips)	Sustained undetectable viral load <50 copies/mL
Number (%)	76 (10.3)	659 (89.7)
Age (years)	44.9	42.5
Male/Female	54/22	457/202
Baseline HIV RNA log_10_ (IQR)	4.85 (4.26–5.21)	4.55 (3.93–5.00)
AIDS (%)	12 (15.8)	59 (9.0)
HBV co-infection (%)	1 (1.3)	30 (4.6)
HCV co-infection (%)	7 (9.2)	59 (9.0)

**Fig. 3:**
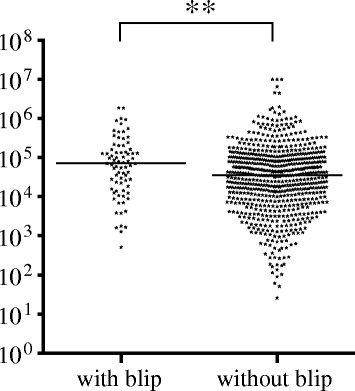
Pre-treatment viral load in subjects with and without viral blips. Line indicates median log10 HIV RNA

Baseline plasma HIV RNA levels were significantly higher in patients with viral blips than in patients with sustained viral suppression, 4.85 log_10_ and 4.55 log_10_ copies/mL, respectively (*p* < 0.01) (Fig. 3).

Among the 659 patients who were suppressed during the entire study period, 481 patients (73 %) had a continuous viral load under the detection threshold (HIV RNA <40/20 copies/mL depending on assay version) after the initial six months. Two hundred and thirty-four patients (32 %, 366 samples) had at least one plasma HIV RNA measurement between 20 and 50 copies/mL. A significantly larger percentage of these samples were found in the group with viral blips, 111 out of 603 (18.4 %), compared with 255 out of 3453 (7.4 %) samples collected from suppressed patients (*p* < 0.0001). Twenty-seven patients developed viral failure, 55.6 % due to consecutive samples > 50 copies/mL more then 6 weeks apart and 44.6 % due to a single HIV RNA > 500 copies/mL. The median HIV RNA among the last group was 1740 (range 662–12900) copies/mL. A significant correlation was observed between viral failure and viral blips (*p* = 0.005). The 27 patients with HIV RNA > 500 copies/mL or 2 consecutive samples 50–500 copies/mL did not have a significant higher baseline viral load. Twenty-six of the 27 patients with viral failure were subsequently re-suppressed, in 77 % without treatment alteration. One patient was lost to follow-up after leaving the country.

As expected, subjects with transient plasma viremia had been sampled more frequently, in median nine times compared with five times in suppressed subjects (*p* < 0.0001).

**Fig. 4:**
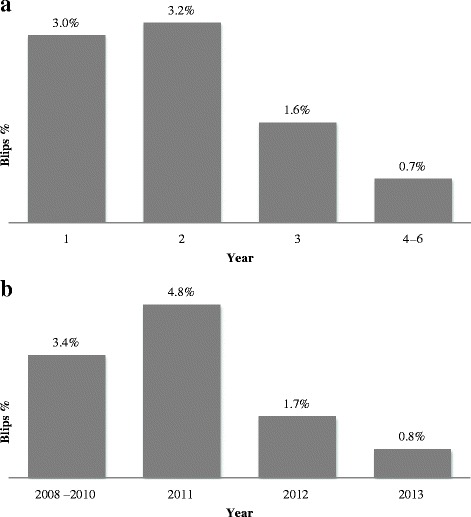
Columns represent the percentage of HIV RNA samples 50–500 copies/mL in comparison to the total number of HIV RNA analysed per year since data collection started (**a**) and in comparison to calendar year (**b**). The first sample in each subject is removed since it is below 50 copies/mL by inclusion criteria

Transient viremia with HIV RNA between 50 and 500 copies/mL was seen in 90 out of 4449 samples (2.0 %). There was a significant difference in blip incidence when comparing different years after ART initiation, with the highest incidence during the second year of observation (Fig. 4a). There was, however, an even more significant difference when comparing calendar years with a peak in 2011 (Fig. 4b). Calendar year and year after initiation are by necessity confounded and when both are entered as predictors in the statistical model only calendar year remains significant (*p* = 0.0001).

There was no significant difference between subjects with and without blips with respect to sex, AIDS-defining diagnoses, co-infection with hepatitis B and/or C viruses, number of primary drug resistance-associated mutations or where they were treated.

Fifty-one per cent of the whole study population was on a regimen containing a non-nucleoside reverse transcriptase inhibitor (NNRTI) and 42 % on a regimen containing a ritonavir-boosted protease inhibitor (PI/r) (Table 2). Only 0.5 % of the samples were registered while the patients were on a dual NNRTI-PI/r-therapy. As dual NRTI backbone, TDF/FTC was used in 66 % of the registered samples and ABC/3TC in the remaining one third.

**Table 2:** HIV RNA levels and ART

	HIV RNA <20 copies/mL	HIV RNA 20–50 copies/mL	HIV RNA 50–500 copies/mL	Viral failure
Number of HIV-RNA samples (%)	3966 (89.1)	366 (8.2)	90 (2.0)	27 (0.6)
Blip HIV RNA copies/ml [median (IQR)]	-	-	76 (55.3–137.8)	-
NNRTI (%)	2083 (52.5)	163 (44.5)	33 (36.7)	5 (18.5)
PI (%)	1625 (41.0)	175 (47.8)	51 (56.7)	21 (77.8)
Others (%)	258 (6.5)	28 (7.7)	6 (6.7)	1 (3.7)

The percentage of patients with blips on a PI/r-based regimen was significantly higher than for subjects on a NNRTI-based regimen, 2.8 % and 1.4 %, respectively (*p* = 0.007 in the logistic model). The same pattern was seen in patients with viral failure where the use of PI/r was significantly more common (*p* = 0.001).

Variables from univariate analyses with *p-*values < 0.1 were entered in the multivariable analysis. This analysis showed that year of measurement, choice of treatment regimen, and baseline VL were all predictors for viral blips (Table 3).

**Table 3:** Uni- and multivariable analyses

	Univariable		Multivariable	
	OR (95 % CI)	*p*	OR (95 % CI)	*p*
Year		<0.001		<0.001
2013	1		1	
2012	2.2 (0.9–5.3)	0.07	2.1 (0.9–5.1)	0.09
2011	6.4 (3.0–14)	<0.001	6.2 (2.9–13)	<0.001
2010	3.7 (1.6–8.5)	0.002	3.5 (1.5–8.1)	0.003
Treatment		0.03		0.057
NNRTI	1		1	
PI	1.9 (1.2–3.1)	0.007	1.8 (1.1–2.8)	0.017
Other	1.4 (0.6–3.2)	0.4	1.3 (0.6–3.0)	0.5
Baseline VL (log_10_ HIV RNA)	1.4 (1.1–1.8)	0.007	1.4 (1.1–1.8)	0.005
CD4 nadir	1.0 (1.0–1.0)	0.14		
Age (years)	1.0 (0.99–1.02)	0.6		
Female	0.8 (0.5–1.4)	0.5		
HBV	0.6 (0.1–3.9)	0.6		
HCV	1.5 (0.6–3.3)	0.4		
AIDS	1.3 (0.7–2.3)	0.4		
Clinic		0.8		
Ethnicity		0.8		
Transmission route		0.6		

## Discussion

Viral blips are defined as transient episodes of detectable viremia in patients on suppressive cART within a given timeframe. There is no generally accepted definition of the upper limit of a viral blip. We defined viral blips as plasma HIV RNA between 50 and 500 copies/mL within a time period of six weeks. The upper limit of 500 copies/mL was chosen because viral loads above this level have been associated with treatment failure [19, 27]. Some studies have, however, reported an increased risk of viral failure with transient viremia with a magnitude less than 500 copies/mL [6, 26] and others found no correlation at all between viral failure and the magnitude of the blips [23]. The upper limit of viral blips in various studies varies from 400 to 2000 HIV RNA copies/mL. Viral loads higher than the upper limit defining a viral blip have usually been considered viral failures [28]. In clinical practise, it is of course very important to distinguish between harmless viral blips and potentially harmful virological failure, where the latter most likely will lead to a change in ART regimen.

In addition to the level of viremia when determining what is a blip or not, there is also the time aspect. When elevated viral loads are found in two consecutive samples drawn within a short timeframe, they are most likely part of a single blip [29]. To separate viral blips from emerging virological failure we chose an interval of six weeks to be considered as a solitary blip. We hypothesized that this would be an appropriate time for a blip to emerge and subside.

The different definitions of viral blips and use of different quantification assays make comparisons between studies of viral blips difficult. Thus, when viral blips in the range 50–500 copies/mL were quantified with three different methods (Versant HIV-1 RNA 1.0 kPCR (Siemens), Abbot Realtime HIV-1, and COBAS Ampliprep/COBAS TaqMan HIV-1 v 2.0 (Roche) assays), only 19 % of the detected viral blips could be reproduced with any of the other methods [30].

We found an incidence of viral blips of 10 % in our Swedish cohort. Older studies have found an incidence of 33–40 % using assays with higher quantification limits [13, 14]. In a more recent study where the same quantification method as in our study was used, but with the upper limit of a viral blip defined as 200 copies/mL, the incidence of blips was 38.1 % [15]. If the same definition was applied to our material, only 69 patients (9.4 %) would have had registered blips. Another recent study reported blip incidence of 13 % but since they used different viral load assays a direct comparison is not possible [16]. The differences between our and other results are probably in part due to differences in frequency of plasma viral load sampling, which was not presented in the latter study. More frequent sampling increases the probability of detecting a viral blip and patients with viral blips are in addition more likely to be sampled more often, at least in close proximity to a viral blip. It can therefore be helpful to not only analyse the number of patients with blips, but also the proportion of blood samples containing blips [28]. One study using the older reverse transcriptase PCR assay with 50 copies/mL as lower limit of detection found viral blips in 3.6 % of the blood samples [8]. The same assay was used in another study where they separated patients with primary- from chronic infection and found ratios of 6.0 % and 13.3 %, respectively [31]. The lowest incidences of reported viral blips are between 1.47 % in a high- and 1.64 % in a low/middle-income cohort [32]. It is not possible to draw any conclusions from these results, however, since there is no information about the quantification methods that were used.

Baseline VL was significantly higher in subjects with blips (median 4.85 log_10_ copies/mL) than in suppressed patients (4.55 log_10_ copies/mL). This is in accordance with findings obtained by older quantification assays [27, 31, 33] and from a recent study where different, not specified, viral load assays were used [16]. The correlation between high pre-treatment viral loads and viral blips is perhaps not unexpected. It is well known that ART is effective in suppressing but not eliminating HIV-infection. Almost all patients who are on cART have residual low-level viremia, approximately 1–10 HIV RNA copies/mL plasma [4, 5, 34–36], and the level of residual viremia has been demonstrated to correlate with pre-therapy plasma HIV RNA levels [3, 37, 38]. Even in patients who have been on suppressive ART for more than ten years, there is an increased risk of viral blips in those with high baseline HIV RNA levels [39].

There is strong evidence that persistent low level viremia in patients on ART is derived from reservoirs of long-lived virus-producing cells that are not affected by currently available drugs that target new cycles of viral replication [35, 40–43]. Viral blips were significantly more common in patients on a boosted PI-based regimen than in patients on an NNRTI-based regimen. This is in line with several other reports, but the opposite has also been found [27, 38, 44, 45]. Possible explanations for why viral blips could be more common in patients on PIs are tolerability issues, which may affect adherence and pharmacokinetic aspects including penetration to sanctuary compartments. There can also be a selection bias between PI- and NNRTI-based regimens such that clinicians are inclined to choose a PI-based treatment for patients with advanced disease or where low adherence could be expected. The association between PI and viral blips then serves as a confounder where it is really the non adherence and the secondary possibility of viral replication in sanctuary sites that is the relevant factor.

The origin of viral blips is still uncertain but probably multifactoral. As for low-level residual viremia, it has been hypothesised that blips are caused by virus released from long-lived cell populations. Other theories are that viral blips are caused by continuous viral replication in “sanctuary sites” with suboptimal drug concentrations despite systemic effective ART and latent reservoirs with productively infected CD4^+^ T cells and macrophages with a very low cell turnover speed that occasionally shed virions [34, 46–48].

One of the most important questions is whether there are any consequences of viral blips, particularly development of subsequent viral failure. We found a significant association between viral blips and viral failure (*p* = 0.005) in accordance with another study [49]. Most likely the majority of the detected cases with viral failure in this study represents treatment interruptions due to adherence issues, especially when more then two thirds were subsequently normalised without treatment alteration. We believe that consecutive HIV RNA > 50 copies/mL more likely represent an actual treatment failure than a single HIV RNA > 500 copies/mL. In some cases however the HIV RNA dynamics cause suspicion that the consecutive samples are in fact one single peak despite more than 6 weeks apart. Others with several months between the sampling could be due to recurrent blips where infrequent sampling fail to detect re-suppression between the events.

Blip magnitude > 200 HIV RNA copies/mL in adherent patients has been associated with a higher risk of viral rebound [6]. Although the opposite has been shown in older studies [8, 13, 21, 22, 25, 26] this might be regarded as an indication to monitor patients with viral blips more closely. Since this is not a prospective study, no causality between blips and viral failure can be proven.

Subjects with viral blips in this study also had a significantly higher incidence of HIV RNA levels in the range 20–50 copies/mL compared with those without blips. This might reflect that patients with high baseline HIV RNA levels, and a large viral reservoir, are more prone to release virus from this reservoir now and then, in the range 20–50, as well as in the range 50–500 copies/mL. It might, however, also be due to overall infrequent sampling, reflecting that the values between 20 and 50 copies/mL are actually the start or tail of missed blips. This low-level viremia has no clinical importance that we know today, but some experts advocate that HIV RNA levels less than 20 copies/mL should be the new cART virological goal based on a study where a correlation between low level viremia and virological rebound was found.

Patients initiating ART are more closely monitored during the first year on treatment than later on. Patients with unexpected increases in plasma viral loads are sampled more frequently in order to determine the nature and course of the viremia. Mapping the natural course of a viral blip would require frequent, perhaps daily, blood sampling of patients, which for obvious reasons is not done in clinical practice. In a patient with unexpected viremia, a new viral load is usually checked within one to three months depending on the magnitude of the increase. By the time of the subsequent blood sampling, the viral load is often undetectable again. Blood sampling as often as every 2–3 days of patients with blips has been performed in one study with 10 patients [8]. Nine out of ten participants had at least one blip during the 3–4 months the study was conducted. These results are different from other studies but they represent a limited population from a short observation time and it has been suggested that they represent stochastic statistical and biological variations [28].

Our study has some limitations. First of all, it is a retrospective study and sampling has therefore been based on clinical guidelines and decisions. Patients are asked about adherence at every visit to the clinics, but data on adherence has not been documented in a standardized way and antiretroviral drug concentrations have not been measured. Poor adherence has been associated with more frequent viral blips in one study [25], but not in others [8, 16, 50]. It is not unlikely that patients missing some doses could have suboptimal plasma antiretroviral drug concentrations which could lead to subsequent viral replication, but poor adherence is probably not the only explanation for viral blips as noted in in a recent study from a highly adherent population [16].

The highest percentage of viral blips was found during the second year after ART initiation (3.2 %). In the multivariat analysis the strong correlation between viral blips and year on treatment is caused by calendar year, which was unexpected. A prior study demonstrated that switching from COBAS TaqMan v1.0 to v2.0, as was the case in this study, resulted in a increased number of detected HIV RNA levels that the researchers concluded was the result of increased sensitivity due to reduced underquantification [51]. Upgrading to COBAS Taqman v2.0 thus theoretically would increase the number of reported blips and not the opposite.. PCR master mix batches are known to have some inter-variability but since they are regularly switched and compared to a kit independent control they are unlikely the source of decreasing blip incidence. Treatment guidelines did not undergo any radical changes during these years and there has not been a substantial change in our patient population. One contributing factor to the decreasing frequency of viral blips could be the overall improved treatment results that we see in Sweden and in other countries. In a review, Fung et al showed that studies based on clinical data tended to have more frequent sampling during the first part of the trials with the consequence that possible blips are missed later because of limited testing [28].

## Conclusions

The Swedish national HIV-cohort has a low incidence of viral blips (10 %). Blips were associated with high baseline viral load and an increased risk of subsequent virological failure. More data is needed to determine the clinical relevance of this phenomenon and further studies would benefit from harmonised definitions.

## Abbreviations

AIDS, acquired immune deficiency syndrome; cART, combination antiretroviral therapy; HBV, hepatitis B virus; HCV, hepatitis C virus; HIV, human immunodeficiency virus; NNRTI, non-nucleoside reverse transcriptase inhibitor; PI, protease inhibitor; VL, viral load
